# Prognostic evaluation of HCC patients undergoing surgical resection: an analysis of 8 different staging systems

**DOI:** 10.1007/s00423-020-02052-1

**Published:** 2020-12-09

**Authors:** Jan Bednarsch, Zoltan Czigany, Daniel Heise, Katharina Joechle, Tom Luedde, Lara Heij, Philipp Bruners, Tom Florian Ulmer, Ulf Peter Neumann, Sven Arke Lang

**Affiliations:** 1 Department of Surgery and Transplantation, University Hospital RWTH Aachen, Pauwelsstrasse 30, 52074 Aachen, Germany; 2 Department of Medicine III, University Hospital RWTH Aachen, Aachen, Germany; 3 Institute of Pathology, University Hospital RWTH Aachen, Aachen, Germany; 4 Department of Radiology, University Hospital RWTH Aachen, Aachen, Germany; 5 Department of Surgery, Maastricht University Medical Centre (MUMC), Maastricht, Netherlands

**Keywords:** HCC, Staging system, ITA.LI.CA, CLIP, Liver resection

## Abstract

**Purpose:**

No consensus exists regarding the most appropriate staging system to predict overall survival (OS) for hepatocellular carcinoma (HCC) in surgical candidates. Thus, we aimed to determine the prognostic ability of eight different staging systems in a European cohort of patients undergoing liver resection for HCC.

**Methods:**

Patients resected for HCC between 2010 and 2019 at our institution were analyzed with Kaplan-Meier and Cox regression analyses. Likelihood ratio (LR) *χ*^2^ (homogeneity), linear trend (LT) *χ*^2^ (discriminatory ability), and Akaike Information Criterion (AIC, explanatory ability) were used to determine the staging system with the best overall prognostic performance.

**Results:**

Liver resection for HCC was performed in 160 patients. Median OS was 39 months (95% confidence interval (CI): 32–46 months) and median RFS was 26 months (95% CI: 16–34 months). All staging systems (BCLC, HKLC, Okuda, CLIP, ITA.LI.CA staging and score, MESH, and GRETCH) showed significant discriminatory ability regarding OS, with ITA.LI.CA score (LR *χ*^2^ 30.08, LT *χ*^2^ 13.90, AIC 455.27) and CLIP (LR *χ*^2^ 28.65, LT *χ*^2^ 18.95, AIC 460.07) being the best performing staging systems.

**Conclusions:**

ITA.LI.CA and CLIP are the most suitable staging system to predict OS in European HCC patients scheduled for curative-intent surgery.

## Introduction

Hepatocellular carcinoma (HCC) is a major global health burden being the third most common cause of cancer-associated mortality worldwide [1, 2]. In the majority of patients, HCC arises on a background of chronic liver disease. Hence, liver transplantation is often considered treatment of choice since it addresses both the underlying liver and the oncological disease [3]. However, a large proportion of HCC patients is too old for transplantation, has major comorbidities, or presents with other contraindications, e.g., active alcoholism as well as advanced tumor stages precluding this approach. Moreover, the limited availability of liver grafts which result in strict allocation regulation and the excellent oncological outcome of surgery in small, solitary HCC underline the importance of liver resections as a major therapeutic option in patients suffering from HCC [4, 5]. Despite recent advances in HCC surgery including the use of dynamic liver function tests, e.g., LiMAx (maximum liver function capacity) or indocyanine green (ICG) and the increasing implementation of minimally invasive liver surgery, a significant proportion of patients is usually regarded as not ideal candidates for surgery based on a high risk of post-hepatectomy liver failure or poor overall oncological prognosis [6–11]. Therefore, identifying preoperative characteristics associated with a higher perioperative risk and prognostic value for oncological outcome has been in the center of interest in HCC. The latter has led to the development of various staging systems aiming to support clinical decision-making in HCC patients.

The most widespread preoperative staging systems are the Milan criteria and the Barcelona Clinic Liver Cancer (BCLC) staging system. While the Milan criteria is used to predict the outcome in HCC undergoing transplantation based on radiologic features, the BCLC system stratifies patients based on radiologic features, physical performance, and liver function. Hence, BCLC is commonly adopted in therapy guidelines, giving distinct recommendations regarding the treatment of choice for each subgroup of patients [12, 13]. In particular, BCLC allocates patients with early stage tumors to curative-intent surgery, while more oncological progressed individuals or patients with impaired liver function are scheduled to interventional or systemic therapy [12]. This traditional paradigm has been challenged by recent reports indicating a survival benefit of liver resection over other treatment modalities regardless of the pre-hepatectomy BCLC stage [14, 15]. Subsequently, various other staging systems have been proposed to overcome limitations of the BCLC staging systems, e.g. ,Cancer of Liver Italian Program (CLIP) score, Groupe d’Etude et de Traitément du Carcinome Hepatocellulaire (GRETCH) score, Italian Liver Cancer (ITA.LI.CA) tumor staging and score, Hong Kong Liver Cancer (HKLC) staging and score, model to estimate survival in hepatocellular carcinoma (MESH) score, or Okuda staging (Table 1) [12, 13, 16–21].

**Table 1:** Synopsis of staging systems for hepatocellular carcinoma

Staging system	Radiologic characteristics				Patient characteristics		Laboratory characteristics				
	Nodule size and/or count or tumor burden	Vascular invasion	Metastasis	Ascites	Performance status	Encephalopathy	AFP	Albumin	Bilirubin	PT/INR	AP
Milan	**x**	**x**	**x**								
BCLC	**x**	**x**	**x**	**x**	**x**	**x**		**x**	**x**	**x**	
HKLC	**x**	**x**	**x**	**x**	**x**	**x**		**x**	**x**	**x**	
Okuda	**x**			**x**				**x**	**x**		
CLIP	**x**	**x**		**x**		**x**	**x**	**x**	**x**	**x**	
ITA.LI.CA staging	**x**	**x**	**x**								
ITA.LI.CA score	**x**	**x**	**x**	**x**	**x**	**x**	**x**	**x**	**x**	**x**	
MESH	**x**	**x**	**x**	**x**	**x**	**x**	**x**	**x**	**x**	**x**	**x**
GRETCH		**x**			**x**		**x**		**x**		**x**

The included characteristics of each staging system are displayed [12, 13, 16–21]. Milan criteria were included for reference reasons. *AFP* alpha-fetoprotein, *AP* alkaline phosphatase, *BCLC* Barcelona Clinic Liver Cancer, *CLIP* Cancer of Italian Liver Program, *GRETCH* Groupe d’Etude et de Traitément du Carcinome Hepatocellulaire, *ITA.LI.CA* Italian Liver Cancer, *HKLC* Hong Kong Liver Cancer, *MESH*, model to estimate survival in hepatocellular carcinoma

Given the variety of staging systems, we aimed to evaluate the prognostic ability of each staging systems to determine the “best” performing model in a European cohort of patients undergoing curative-intent surgery for HCC.

## Material and methods

### Patients

The study comprised one hundred sixty (*n* = 160) consecutive HCC patients who underwent curative-intend surgery at the University Hospital RWTH Aachen (UH-RWTH) between 2010 and 2019. Clinical staging was performed according to international guidelines, and all individuals had localized tumors without signs of systemic disease. The study was conducted at the UH-RWTH in accordance with the requirements of the Institutional Review Board of the RWTH-Aachen University (EK 115/20), the current version of the Declaration of Helsinki, and the good clinical practice guidelines (ICH-GCP).

### Staging and surgical technique

All patients who were referred for surgical treatment to our institution underwent a detailed clinical work-up as previously described [2, 4]. Therefore, the number, size, and location of tumor nodules as well as the presence of distant metastases were evaluated by magnetic resonance imaging (MRI) or computed tomography (CT). The preoperative risk assessment was carried out based on the American Society of Anesthesiologists (ASA) and the Eastern Cooperative Oncology Group (ECOG) performance status, calculation of the future liver remnant (FLR), as well as parenchymal liver function as assessed by standard laboratory parameters and the LiMAx test (Humedics® GmbH, Berlin, Germany) [22]. Non-invasive liver function tests were routinely carried out, but no preoperative liver biopsies were obtained to assess the quality of the liver parenchyma. Patients staged BCLC A to BCLC C without any evidence of extrahepatic spread as well as compensated liver function were considered candidates for surgery as primary treatment. The definitive decision for hepatectomy was made by a staff hepatobiliary surgeon and approved by the institutional interdisciplinary tumor board in every patient. Liver resection was carried out in accordance with common clinical standards [2, 4]. In brief, an intraoperative ultrasound was performed to visualize the local tumor spread and other suspicious lesions. The decision for either anatomic resections or non-anatomic atypical wedge resections with an adequate resection margin was based on the surgeon’s preference. Parenchymal transection was carried out using the Cavitron Ultrasonic Surgical Aspirator (CUSA®, Integra LifeSciences®, Plainsboro NJ, USA) with low CVP and intermittent Pringle maneuvers if necessary in open hepatectomy. In laparoscopic hepatectomy, parenchymal transection was commonly performed by Thunderbeat® (Olympus K.K., Tokyo, Japan), Harmonic Ace® (Ethicon Inc. Somerville, NJ, USA), or laparoscopic CUSA (Integra LifeSciences, NJ, USA) in combination with vascular staplers (Echelon, Ethicon, Somerville, NJ, USA) or polymer clips (Teleflex Inc., PA, USA). The anesthesiologic management was based on a restrictive fluid intervention strategy ensuring a low central venous pressure (CVP) during parenchymal dissection.

### Statistical analysis

The primary endpoint of this study was to identify the staging system with the best prognostic ability for OS. Overall performance was defined by homogeneity (small differences in OS among patients within the same stage), discriminatory ability (great differences in OS among patients within different stages), and monotonicity of gradients (longer OS in patients in earlier stages than in more advanced stages within the same system) as previously described [23]. Therefore, Cox regression models of each staging systems were established and subsequently used to calculate the likelihood ratio (LR) *χ*^2^ to determine homogeneity, linear trend (LT) *χ*^2^ to assess discriminatory ability, and both LR *χ*^2^ and LT *χ*^2^ to measure monotonicity of gradients as well as Akaike Information Criterion (AIC) to describe the explanatory ability of the particular staging system [23]. The degrees of freedom were set to 1 in all calculations to allow the comparison of prognostic systems with a different total number of stages. Higher LR *χ*^2^ and LT *χ*^2^ as well as lower AIC indicate a better fitting model to predict OS in this statistical approach. Milan criteria were included in the analysis for reference reasons. Data derived from continuous variables are presented as median and interquartile range. Survival curves were generated by the Kaplan-Meier method and compared with the log-rank test. Median follow-up was accessed with the reverse Kaplan-Meier method. Complications are reported as in-hospital morbidity and in-hospital mortality. Perioperatively deceased patients were included in all survival analyses. The level of significance was set to *p* < 0.05, and *p* values are given for two-sided testing. Analyses were performed using SPSS Statistics 24 (IBM Corp., Armonk, NY, USA).

## Results

### Preoperative, operative, and postoperative data

A total of 160 patients with a median age of 68 years and median body mass index (BMI) of 26 kg/m^2^ who underwent curative-intent surgery for HCC at our institution from 2011 to 2019 were included in this study with more than half of the study cohort (60.0%, 96/160) belonging to the performance status category ASA III or higher. The vast majority of the patients were categorized as Child-Pugh A (93.1%, 149/160) with a median Child-Pugh score (CPS) of 5 and a median model for end-stage liver disease (MELD) score of 6. The median nodule count was 1 (range 1–7), and the median largest tumor diameter is 55 mm (range 6–228 mm). A tumor burden > 50% was detected in 5.0% (8/160) of the patients, while an overall invasion to major vessels was observed in 26.3% (42/160). Of all patients, 3.1% (5/160) underwent transarterial chemoembolization (TACE) and 1.3% (2/160) transarterial radioembolization (TARE) prior to surgery. A minority of the individuals treated for HCC (27.5%, 44/160) underwent laparoscopic liver resection, and the median operative time was 207 min. R0 resection was achieved in 95.6% (153/160) of the patients. Median hospital stay was 9 days. No complications were detected in 47% (75/160) of the patients. In contrast, 46 patients (28.8%) experienced major postoperative complications (Clavien-Dindo ≥ 3) and 10 patients (6.3%) deceased perioperatively. More clinicopathological and perioperative characteristics are outlined in Table 2, and a detailed overview of the applied staging systems (Milan criteria, BCLC, HKLC, Okuda, CLIP, ITA.LI.CA staging, ITA.LI.CA score, MESH, and GRETCH) is presented in Table 3.

**Table 2:** Clinical and perioperative characteristics

Demographics	
Gender, m/f (%)	115 (71.9) / 45 (28.1)
Age (years)	68 (60–75)
BMI (kg/m^2^)	26 (23–29)
Preoperative PVE, *n* (%)	8 (5.0)
Preoperative TACE, *n* (%)	5 (3.1)
Preoperative TARE, *n* (%)	2 (1.3)
Preoperative TACE and TARE, *n* (%)	1 (0.6)
ASA, *n* (%)	
I	3 (1.9)
II	61 (38.1)
III	91 (56.9)
IV	5 (3.1)
V	0
Liver disease, *n* (%)	
ALD	37 (23.1)
NAFLD	66 (41.3)
Viral	43 (26.9)
Cryptogenic/others	14 (8.8)
Preoperative liver function	
MELD score	6 (6–6)
AFP (ng/ml)	9 (3–93)
Albumin (g/dl)	4.0 (3.6–4.4)
AST (U/l)	42 (30–62)
ALT (U/l)	35 (23–56)
GGT (U/l)	101 (52–205)
Total bilirubin (mg/dl)	0.52 (0.40–0.80)
Platelet count (/nl)	221 (170–282)
Alkaline Phosphatase (U/l)	99 (73–139)
Prothrombine time (%)	93 (85–100)
INR	1.04 (0.98–1.10)
Creatinine (mg/dl)	0.85 (0.70–1.04)
Hemoglobin (g/dl)	13.3 (11.7–14.6)
Child-Pugh, *n* (%)	
A	149 (93.1)
B	11 (6.9)
C	0
Child-Pugh score	5 (5–5)
Preoperative imaging features	
Number of nodules	1 (1–2)
Largest nodule diameter (mm)	55 (38–81)
Tumor burden > 50%, *n* (%)	8 (5.0)
Overall macrovascular invasion, *n* (%)	42 (26.3)
Portal vein invasion, *n* (%)	27 (16.9)
Extrahepatic vascular invasion, *n* (%)	11 (6.9)
Portal vein thrombosis, *n* (%)	8 (5.0)
Ascites, *n* (%)	6 (3.8)
Operative data	
Laparoscopic resection, *n* (%)	44 (27.5)
Conversation rate, *n* (%)	4 (9.1)
Operative time (minutes)	207 (146–270)
Operative procedure, *n* (%)	
Atypical	51 (31.9)
Segmentectomy	24 (15.0)
Bisegmentectomy	13 (8.1)
Hemihepatectomy	42 (26.3)
Extended liver resection	24 (15.0)
Other	6 (3.8)
Additional procedures (RFA, etc.), *n* (%)	7 (4.3)
Pringle maneuver, *n* (%)	8 (5.1)
Duration of Pringle maneuver (min)*	20 (13–24)
Intraoperative blood transfusion, *n* (%)	49 (31.6)
Intraoperative FFP, *n* (%)	69 (44.5)
Intraoperative platelet transfusion, *n* (%)	4 (2.6)
Pathological examination	
R0 resection, *n* (%)	153 (95.6)
T category, *n* (%)	
T1/T2	119 (76.3)
T3/T4	37 (23.7)
Microvascular invasion, *n* (%)	71 (49.0)
Tumor grading, *n* (%)	
G1/G2	128 (80.5)
G3/G4	31 (19.5)
Postoperative data	
Intensive care stay, days	1 (1–1)
Hospitalization, days	9 (6–15)
Postoperative complications, *n* (%)	
No complications	75 (46.9)
Clavien-Dindo I	20 (12.5)
Clavien-Dindo II	19 (11.9)
Clavien-Dindo IIIa	18 (11.3)
Clavien-Dindo IIIb	9 (5.6)
Clavien-Dindo IVa	7 (4.4)
Clavien-Dindo IVb	2 (1.3)
Clavien-Dindo V	10 (6.3)
PHLF 50-50 criteria*, *n* (%)	2 (1.3)
PHLF ISGLS*, *n* (%)	36 (22.5)
ISGLS grade, *n* (%)	
A	24 (66.7)
B	5 (13.9)
C	7 (19.4)
Postoperative blood transfusion	29 (18.7)
Postoperative FFP	13 (8.4)
Postoperative platelet transfusion	4 (2.6)
Follow-up data	
Recurrence-free survival (months)	26 (16–34)
Overall survival (months)	39 (32–46)
Liver transplantation, *n* (%)	2 (1.3%)

Data presented as median and interquartile range if not noted otherwise. Follow-up data is presented as median and 95% CI. *Postoperative liver failure was assessed by the 50-50-criteria and the ISGLS definition [11, 24]. *ALD* alcoholic liver disease, *ALT* alanine aminotransferase, *ASA* American Society of Anesthesiologists classification, *AST* aspartate aminotransferase, *BCLC* Barcelona Clinical Liver Cancer staging system, *BMI* body mass index, *CI* confidence interval, *FFP* fresh frozen plasma, *GGT* gamma glutamyltransferase, *INR* international normalized ratio, *ISGLS* International Study Group of Liver Surgery, *MELD* model of end-stage liver disease, *MWA* microwave ablation, *NAFLD* non-alcoholic fatty liver disease, *PHLF* post-hepatectomy liver failure, *PVE* portal vein embolization, *TACE* transarterial chemoembolization, *TARE* transarterial radioembolization

**Table 3:** Staging systems for HCC guiding clinical management and predicting survival

Milan criteria	*n* **(%)**	Median OS (95% CI)	*p* value
Yes	48 (30.0)	58 (24–92)	.012
No	112 (70.0)	31 (22–40)	
BCLC	*n* (%)	Median OS (95% CI)	*p* value
0*	6 (3.8)	62 (40–85)	.001
A	89 (55.6)	55 (32–78)	
B	38 (23.8)	23 (9–37)	
C	27 (16.9)	15 (4–22)	
D	0	n.a.	
HKLC	*n* (%)	Median OS (95% CI)	*p* value
I	56 (35.0)	48 (27–69)	.001
IIa	2 (1.3)	n.a.	
IIb	51 (31.9)	66 (44–88)	
IIIa	7 (4.4)	3 (0–8)	
IIIb	33 (20.6)	20 (11–29)	
IVa	11 (6.9)	38 (13–61)	
IVb–Vb	0	n.a.	
Okuda staging	*n* (%)	Median OS (95% CI)	*p* value
I	138 (86.3)	42 (28–56)	.001
II	22 (13.8)	12 (6–18)	
III	0	n.a.	
CLIP	*n* (%)	Median OS (95% CI)	*p* value
0*	57 (50.4)	91 (68–114)	.001
1	42 (37.2)	38 (12–64)	
2	7 (6.2)	15 (4–26)	
3	6 (5.3)	3 (0–9)	
4	1 (0.9)	n.a.	
5–6	0	n.a.	
ITA.LI.CA staging	*n* (%)	Median OS (95% CI)	*p* value
0*	6 (3.8)	63 (40–85)	.016
A	41 (25.6)	58 (33–83)	
B1	52 (32.5)	41 (23–59)	
B2	15 (9.4)	33 (0–72)	
B3	35 (21.9)	20 (13–27)	
C	11 (6.9)	38 (13–63)	
ITA.LI.CA score	*n* (%)	Median OS (95% CI)	*p* value
0	1 (0.9)	n.a.	.001
1	10 (8.8)	42 (36–48)	
2	27 (23.9)	130 (22–238)	
3	21 (18.6)	41 (0–84)	
4	22 (19.5)	38 (0–77)	
5	18 (15.9)	17 (9–25)	
6	3 (2.7)	n.a.	
7	7 (6.2)	8 (5–11)	
8	3 (2.7)	6 (0–12)	
9	1 (0.9)	2 (0–2)	
10–13	0	n.a.	
MESH	*n* (%)	Median OS (95% CI)	*p* value
0*	19 (16.8)	88 (63–114)	.001
1	31 (27.4)	41 (16–66)	
2	40 (35.4)	41 (0–82)	
3	17 (15.0)	21 (2–40)	
4	4 (3.5)	10 (3–17)	
5*	2 (1.8)	3 (2–3)	
6	0	n.a.	
GRETCH	*n* (%)	Median OS (95% CI)	*p* value
0	57 (50.4)	55 (26–83)	.001
1	9 (8.0)	16 (9–22)	
2	31 (27.4)	20 (7–33)	
3	12 (10.6)	10 (4–16)	
4	3 (2.7)	n.a.	
5	1 (0.9)	n.a.	
6–11	0	n.a.	

Milan criteria were included for reference reasons. *Mean. Survival data is presented in months. The log-rank test was carried out for each staging system. *BCLC* Barcelona Clinic Liver Cancer, *CLIP* Cancer of Liver Italian Program, *GRETCH* Groupe d’Etude et de Traitément du Carcinome Hepatocellulaire, *ITA.LI.CA* Italian Liver Cancer, *HKLC* Hong Kong Liver Cancer, *MESH* model to estimate survival in hepatocellular carcinoma, *OS* overall survival

### Survival analysis

**Fig. 1:**
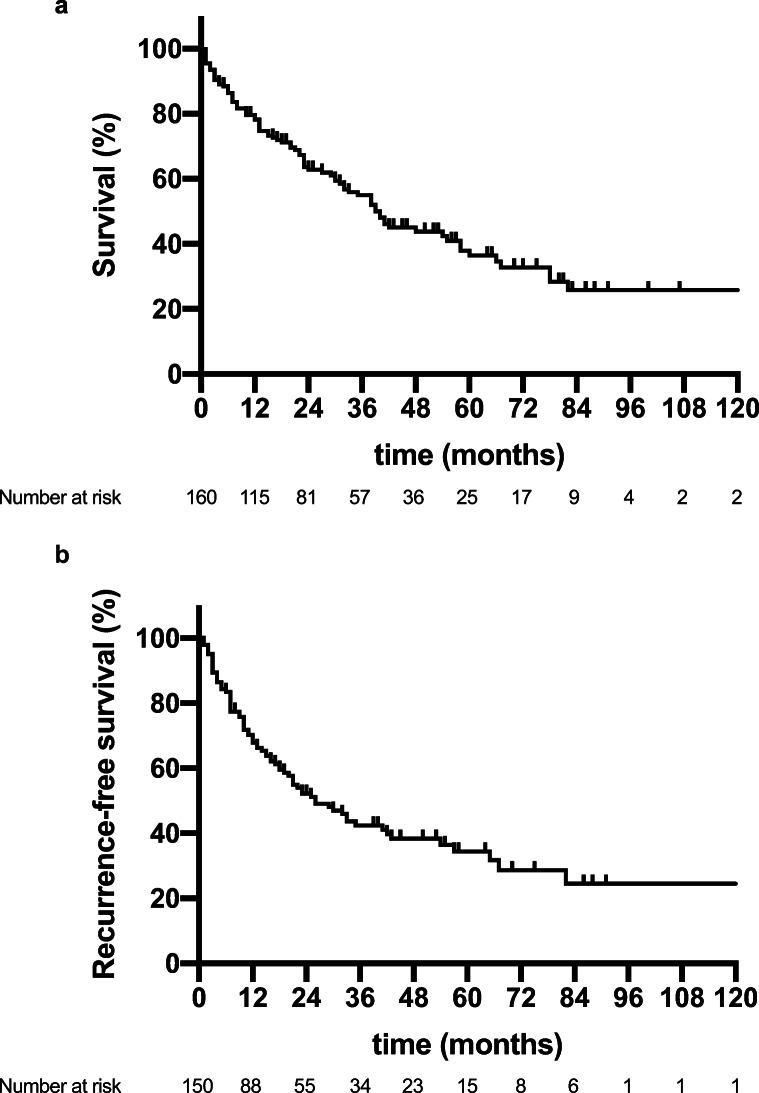
Oncological survival in hepatocellular carcinoma of the study cohort. **a** Overall survival in hepatocellular carcinoma. The median OS of the cohort was 39 months (95% CI: 32–46 months). **b** Recurrence-free survival in hepatocellular carcinoma. The median RFS of the cohort was 26 months (95% CI: 16–34 months). OS, overall survival; RFS, recurrence-free survival

**Fig. 2:**
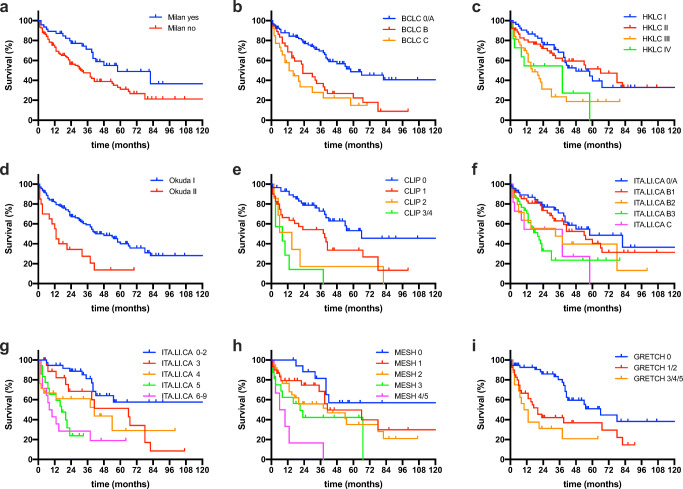
Oncological survival in hepatocellular carcinoma stratified by different staging systems. **a** Overall survival stratified by Milan criteria. Milan criteria were included for reference reasons. Patients fulfilling the Milan criteria showed a median OS of 58 compared to 31 months in patients outside the Milan criteria (*p* = 0.012 log rank). **b** Overall survival stratified by BCLC staging. The mean OS was 63 months for BCLC 0, while the median OS was 55 months for BCLC A, 23 months for BCLC B, and 13 months for BCLC C (*p* = 0.001 log rank). **c** Overall survival stratified by HKLC staging. The median OS was 48 months for HKLC I, 66 months for HKLC II, 15 months for HKLC III, and 38 months for HKLC IV (*p* = 0.001 log rank). **d** Overall survival stratified by Okuda staging. The median OS was 42 months for Okuda I and 12 months for Okuda II (*p* = 0.001 log rank). **e** Overall survival stratified by CLIP. The mean OS for CLIP 0 was 90 months, while the median OS was 38 months for CLIP 1, 15 months for CLIP 2, and 8 months for CLIP 3/4 (*p* = 0.001 log rank). **f** Overall survival stratified by ITA.LI.CA staging. The median OS was 58 months for ITA.LI.CA 0/A, 41 months for ITA.LI.CA B1, 33 months for ITA.LI.CA B2, 20 months for ITA.LI.CA B3, and 38 months for ITA.LI.CA C (*p* = 0.009 log rank). **g** Overall survival stratified by ITA.LI.CA score. The median OS was 130 months for ITA.LI.CA 0–2, 41 months for ITA.LI.CA 3, 38 months for ITA.LI.CA 4, 17 months for ITA.LI.CA 5, and 8 months for ITA.LI.CA 6–9 (*p* = 0.001 log rank). **h** Overall survival stratified by MESH. The mean OS for MESH 0 was 89 months, while the median OS was 41 months for MESH 1, 41 months for MESH 2, 21 months for MESH 3, and 6 months for MESH 4/5 (*p* = 0.001 log rank). **i** Overall survival stratified by GRETCH. The median OS was 55 months for GRETCH 0, 22 months for GRETCH 1/2, and 10 months for GRETCH 3/4/5 (*p* = 0.001 log rank). BCLC, Barcelona Clinic Liver Cancer; CI, confidence interval; CLIP, Cancer of Liver Italian Program; GRETCH, *Groupe d’Etude et de Traitément du Carcinome Hepatocellulaire*. ITA.LI.CA, Italian Liver Cancer; HKLC, Hong Kong Liver Cancer; MESH, model to estimate survival in hepatocellular carcinoma; OS, overall survival

After a median follow-up of 50 months, the median OS of the cohort was 39 months (95% confidence interval (CI): 32–46 months), and the median RFS was 26 months (95% CI: 16–34 months). Further, we conducted multiple secondary survival analyses within the different staging systems. Patients fulfilling the Milan criteria showed a median OS of 58 (95% CI: 24–92 months) compared to 31 months (95% CI: 24–92 months, 22–40 months) in patients outside the Milan criteria (*p* = 0.012 log rank). Regarding BCLC staging system, the median OS was 63 months (95% CI: 40–85 months) for BCLC 0, 55 months (95% CI: 32–78 months) for BCLC A, and 23 months (95% CI: 9–37 months) and 13 months (95% CI: 4–22 months) for BCLC C (*p* = 0.001 log rank). More details regarding OS in different staging systems are shown in Table 3, Figs. 1, and 2.

### Comparative analysis of different staging systems predicting survival

All nine staging systems demonstrated a significant difference of probability of OS when analyzed using Kaplan-Meier analysis (Table 3, Fig. 2). To determine the “best” fitting model, LR *χ*^2^, LT *χ*^2^, and AIC were determined. Due to missing data (alpha-fetoprotein, AFP) which was required for some of the staging systems (CLIP, ITA.LI.CA score, MESH, and GRETCH), the overall cohort (*n* = 160) was analyzed for Milan criteria, BCLC, HKLC, Okuda, and ITA.LI.CA staging and subset of patients (*n* = 113) for all staging systems separately (Table 4).

**Table 4:** Comparison of prognostic stratification of different staging systems

Staging systems	Linear trend *χ*^2^	Likelihood ratio *χ*^2^	Akaike Information Criterion
Sub cohort (*n* = 113)			
ITA.LI.CA score	13.90	30.08	455.27
CLIP	18.95	28.65	460.07
Okuda staging	8.16	19.29	464.53
BCLC	10.25	16.43	466.97
MESH	9.71	15.29	467.40
GRETCH	8.28	14.37	468.91
ITA.LI.CA staging	3.46	8.09	474.19
HKLC	2.25	6.23	475.78
Milan criteria	1.27	1.42	480.30
All patients (*n* = 160)			
BCLC	13.27	20.48	764.21
Okuda staging	7.37	16.21	770.45
ITA.LI.CA staging	7.42	13.09	770.23
HKLC	4.35	10.08	772.89
Milan criteria	4.90	6.21	775.91

Milan criteria were included for reference reasons. Higher likelihood ratio *χ*^2^ and linear trend *χ*^2^ as well as lower AIC indicate a better fitting model to predict survival. *AIC* Akaike Information Criterion, *BCLC* Barcelona Clinic Liver Cancer, *CLIP* Cancer of Liver Italian Program, *GRETCH* Groupe d’Etude et de Traitément du Carcinome Hepatocellulaire, *ITA.LI.CA* Italian Liver Cancer, *HKLC* Hong Kong Liver Cancer, *MESH* model to estimate survival in hepatocellular carcinoma

In the overall cohort, BCLC performed best among the analyzed staging system with a LR *χ*^2^ of 20.48, LT *χ*^2^ of 13.27, and AIC of 764.21 outranking all other staging systems in each criterion. In contrast, Milan criteria showed the lowest LR *χ*^2^ (6.21) and highest AIC (775.91), while HKLC was the staging system with the lowest LT *χ*^2^ (4.35). In the sub cohort, ITA.LI.CA score presented with the highest LR *χ*^2^ (30.08) and lowest AIC (455.27) of all staging systems. The highest LT *χ*^2^ (18.95) was determined for CLIP. Similar to the overall cohort, HKLC (LR *χ*^2^ of 9.23, LT *χ*^2^ of 2.25, and AIC of 475.78) and Milan criteria (LR *χ*^2^ of 1.42, LT *χ*^2^ of 1.27, and AIC of 480.30) were the least fitting models to predict OS. More details regarding the different staging systems are outlined in Table 4.

## Discussion

HCC represents one of the major global health issues with liver resection being the treatment of choice in patients with compensated liver function [1–3]. Given this importance of the disease, a variety of staging systems reflecting oncological prognosis and guiding treatment decisions have been proposed, but no international consensus has been achieved which staging system should be preferred [12, 13, 16–21]. In a European cohort of patients, we were able to demonstrate a superiority of ITA.LI.CA score and CLIP over various other staging systems in their prognostic ability for OS after surgical resection. Our data does further suggest that staging systems incorporating biochemical markers of tumor biology (AFP) provide more solid estimates for OS in surgical patients than staging systems focusing on radiological characteristics only. This suggests ITA.LI.CA score and CLIP as the preferable staging systems for preoperative risk assessment balancing oncological outcome with perioperative risks in patients with HCC scheduled for liver surgery.

The BCLC staging system is traditionally considered to guide treatment decision in European patients and provides the basis of the current guideline of the European Society for Medical Oncology (ESMO) [25]. BCLC assigns early stage HCC in patients with compensated liver function and good performance status to surgical therapy, while more advanced disease stages or more compromised individuals are referred to ablative and locoregional therapies or palliative treatment [12, 13]. However, such conservative interpretation of the BCLC staging has to be viewed critically in the era of modern HCC surgery using minimal invasive techniques and novel liver function tests which resulted in significantly improved patient selection and perioperative outcomes [6–11]. Correspondingly, two multicenter studies have shown that liver resection for HCC patients results in survival benefit over medical or interventional therapy regardless of their BCLC stage [14, 15]. These observations are further reassured by a randomized trial indicating better survival in BCLC B patients undergoing liver resection compared to TACE [26]. Therefore, more patients with higher BCLC stages are nowadays considered candidates for surgery provided that severe liver dysfunction and a significantly impaired performance status are absent [14, 15]. Despite this accepted expansion of the BCLC criteria, there is lacking evidence whether higher BCLC stages—which were originally not developed to predict survival in patients undergoing surgery—can be used to provide a basis for the selection of surgical candidates. Interestingly, our data does suggest significant differences in survival in between patients categorized BCLC 0/A compared to BCLC B/C but less discriminative value of the staging system in BCLC B and C categories (Fig. 2b).

One might argue that for patients undergoing surgery, the pathology-based Union for International Cancer Control (UICC) staging systems provides an excellent stratification for estimated postoperative OS. The TNM staging usually does predict OS well in HCC patients but does not take the underlying liver disease into account which limits its overall prognostic ability [27]. Therefore, some staging systems do incorporate the pathological TNM staging and add individual patient characteristics to overcome this major limitation (e.g., Japanese Integrated System (JIS) or Chinese University Prognostic Index (CUPI)) [28, 29]. However, pathological staging only allows a post hoc assessment and is not available for the preoperative decision-making and patient selection. We therefore decided not to include staging systems requiring data based on postoperative pathological examinations into our present analysis.

Similar to the BCLC system, all of the reported staging systems are originally designed to cover the whole disease spectrum of HCC but not exclusively to predict OS in surgical candidates [12, 13, 16–21]. Furthermore, the initial publications regarding development and validation of the different prognostic staging systems are based on large heterogeneous cohorts using various treatment approaches including palliation. This explains the observation that advanced stages of some staging systems are not represented in our analysis (Table 3, Fig. 2). In addition, previous literature predominantly compares various prognostic scores using patient cohorts with a broad disease spectrum and different treatment modalities and, therefore, might have limited use for the selection and stratification of surgical candidates [30]. Nonetheless, to the best of our knowledge, this analysis is the first report in the literature comparing a plethora of staging systems in a European cohort of HCC patients undergoing curative-intent surgery.

Based on our comparative analysis, we identified the ITA.LI.CA score and CLIP as preferable staging system for patients scheduled for liver resection. CLIP was introduced in 1998 and can be considered an advancement over the older Okuda staging which was published in 1985 [19, 20]. CLIP basically uses the same set of variables as the Okuda staging but added AFP and the presence of portal vein thrombosis to the assessed patient characteristics [20]. Despite being relatively old and simple compared to some more novel staging systems, CLIP performed well against other staging systems in comparative analyses. In particular, CLIP outranked BCLC, HCLC, JIS, GRETCH, CUPI, Okuda staging, and TNM staging in its overall prognostic performance in a large Taiwanese study with 3000 patients [31]. Another study from China also demonstrated a higher prognostic value of CLIP for 3- and 6-month OS compared to other staging systems [32]. Based on the small variable set included into calculating the score (tumor volume compared to liver volume, Child-Pugh category, AFP, and the presence of portal vein thrombosis), CLIP is feasible to be used in surgical candidates and does display decent prognostic ability in our current study. Although showing the best discriminatory ability among the investigated staging systems, CLIP was inferior to the novel ITA.LI.CA score in terms of homogeneity and overall explanatory ability. ITA.LI.CA is a complex system-based ITA.LI.CA staging which stratifies patients with respect to size and number of tumor nodules, vascular invasion, and metastasis into four main and some sub-stages [18]. Interestingly, ITA.LI.CA staging performed inferior to the standard BCLC staging in our analysis. The ITA.LI.CA score utilizes the ITA.LI.CA staging and adds functional status, Child-Pugh score, and AFP to calculate a score ranging from 0 to 13 corresponding to overall prognosis in HCC patients [18]. Of note, ITA.LI.CA score has already been validated with an external cohort of patients in a study analyzing 1500 patients undergoing various treatments and showed prognostic superiority over CLIP, HKLC, JIS, ITA.LI.CA staging, and BCLC [33].

Despite showing the best mathematical abilities to predict survival, ITA.LI.CA and CLIP are certainly not perfect from a theoretical point of view as illustrated by the fact that patients with moderate HCC stages could have inferior OS compared to patients with higher cancer stages especially in the ITA.LI.CA staging (Fig. 2). This underlines that staging systems are helpful regarding patient selection but are just one of multiple characteristics guiding decision-making in this complex disease.

Interestingly, the two staging systems which showed the best prognostic ability (CLIP and ITA.LI.CA score) in our setting were originally developed using European patient cohorts. In contrast, the HKLC staging which is based on Asian HCC patients performed only slightly better than the Milan criteria which we have included in our analysis for reference reasons [13, 21]. These observations may suggest a potential difference between Asian and European patient cohorts and its impact on the prognostic ability of the various staging systems. General disease etiology and even genomic characteristics vary between Asian and European patients [34]. Also, the general approach to HCC seems to be more aggressive in Asian cohorts. This might partially be explained by the larger proportion of viral etiology in Eastern patients which results in a generally younger HCC population with often less severe underlying cirrhosis and fewer comorbidities [35]. Therefore, staging systems developed for European cohorts might be more suitable for European patients. The same is true for Eastern patients as recently demonstrated within a large Singaporean cohort. In a comparative analysis of Selby et al. comprising 716 patients, HKLC showed a better performance in guiding treatment compared to BCLC [36]. These considerations do also imply limitations when the results of comparative analyses of staging systems in Eastern patients are directly transferred to Western HCC patients.

Among the staging systems that do not include AFP to correlate radiological and clinical patient characteristics with tumor biology, BCLC showed good results in our cohort. However, in our subgroup of patients with available information on AFP levels, CLIP and ITA.LI.CA score provided a better overall staging performance. Based on this, AFP seems to be a major contributor for accurate staging of HCC patients undergoing surgery. AFP is a known predictor of OS in various clinical situations of HCC patients and characteristics of the tumor [37]. Thus, it is not surprising that staging systems incorporating AFP might be superior in their overall prognostic performance. This observation does further underline the importance of tumor biology and the individual genetic pathogenesis of HCC. Nault et al. have recently proposed a gene score including 5 genes to predict OS and demonstrated significant prognostic accuracy in a surgical cohort of patients [38]. It is therefore important that future staging systems integrate novel biomarkers to further increase the prognostic value of pretreatment staging in HCC patients.

Like any other clinical study, our analysis has certain inherent limitations. All HCC patients analyzed in this study underwent treatment in a monocentric setting reflecting our individual clinical approach to this particular disease, and the study is based on a retrospective data collection which was not obtained in a controlled prospective clinical trial. This also results in large proportion of ASA III patients and individuals with higher BCLC stages due to our liberal department policy. Further, our data set appears small compared to some other studies especially from Asian cohorts. Most importantly, however, the majority of studies focusing on staging systems for HCC comprise heterogeneous cohorts in which curative treatments are carried out in the minority of patients.

Notwithstanding the aforementioned limitations, we here provide a detailed analysis of a plethora of HCC staging systems in a European cohort of patients who underwent curative-intent liver resection, demonstrating ITA.LI.CA score and CLIP to be the most suitable staging systems for surgical candidates.

## Conclusion

All staging systems assessed showed certain discriminatory ability regarding OS of patients undergoing liver resection for HCC. However, the ITA.Li.CA score and CLIP demonstrated a superior prognostic ability compared to other staging systems in our European cohort.

## Abbreviations

AIC: Akaike Information Criterion
ALD: Alcoholic liver disease
ALT: Alanine aminotransferase
AP: Alkaline phosphatase
ASA: American Society of Anesthesiologists
AST: Aspartate aminotransferase
BCLC: Barcelona Clinic Liver Cancer
BMI: Body mass index
CLIP: Cancer of Liver Italian Program
CPS: Child-Pugh score
CI: Confidence interval
CRP: C-reactive protein
CT: Computed tomography
CUPI: Chinese University Integrated System
CUSA: Cavitron Ultrasonic Surgical Aspirator
CVP: Central venous pressure
DFS: Disease-free survival
ECOG: Eastern Cooperative Oncology Group
ESMO: European Society for Medical Oncology
FFP: Fresh frozen plasma
FLR: Future liver remnant
GCP: Good clinical practice
GGT: Gamma glutamyltransferase
GRETCH: Groupe d’Etude et de Traitément du Carcinome Hepatocellulaire
HCC: Hepatocellular carcinoma
HKLC: Hong Kong Liver Cancer
INR: International normalized ratio
ISGLS: International Study Group of Liver Surgery
ITA.LI.CA: Italian Liver Cancer
JIS: Japanese Integrated System
LR: Likelihood ratio
LT: Linear trend
MELD: Model of end-stage liver disease
MESH: Model to estimate survival in hepatocellular carcinoma
MRI: Magnetic resonance imaging
NAFLD: Non-alcoholic fatty liver disease
OS: Overall survival
PHLF: Post-hepatectomy liver failure
PVE: Portal vein embolization
TACE: Transarterial chemoembolization
TARE: Transarterial radioembolization
UH-RWTH: University Hospital Rheinisch-Westfälische Technische Hochschule
UICC: Union for International Cancer Control
